# Effects of curcumin on dyslipidemia and body mass index in metabolic disorders: a systematic review and meta-analysis

**DOI:** 10.3389/fnut.2025.1655775

**Published:** 2025-10-16

**Authors:** Fang Fang, Defeng Qin, Yong Li, Fang Liu, Jinlin Wu

**Affiliations:** Department of Endocrinology, Chongqing Traditional Chinese Medicine Hospital, Chongqing, China

**Keywords:** curcumin, blood lipid, body mass index, metabolic diseases, meta

## Abstract

**Objective:**

This study aimed to investigate the effects of curcumin on blood lipid levels and body mass index (BMI) in patients with metabolic diseases.

**Methods:**

A systematic database search identified 587 records, from which 11 randomized controlled trials (RCTs) involving 662 participants were included. The analysis evaluated changes in triglycerides (TG), total cholesterol (TC), high-density lipoprotein cholesterol (HDL-C), low-density lipoprotein cholesterol (LDL-C), and BMI. Both fixed-effects models (FEM) and random-effects models (REM) were used for statistical analysis. Funnel plot asymmetry tests (Begg and Egger), Baujat, and Galbraith analyses assessed heterogeneity and potential publication bias. Cochrane RevMan (version 2.0) evaluated the risk of bias.

**Results:**

Curcumin supplementation significantly reduced TG levels [mean difference (MD): −16.76 mg/dL, REM], TC levels (MD: −10.59 mg/dL, FEM), and BMI (MD: −0.94 kg/m^2^ in both models). However, no significant effect was found for HDL-C and LDL-C under the random-effects model, whereas fixed-effects models showed variable results, highlighting the inconsistency and the need for further investigation. For HDL-C, the random-effects model (REM), which accounts for high heterogeneity (I^2^ = 83%), showed no significant change (MD: −1.90 mg/dL, *p* = 0.11), while the fixed-effects model (FEM) indicated a modest increase. Due to substantial between-study variability, the REM results are more reliable and suggest no consistent effect of curcumin on HDL-C levels. For LDL-C, the random-effects model (REM), which accommodates high heterogeneity (I^2^ = 67%), showed no significant reduction (MD: 5.01 mg/dL, *p* = 0.12), whereas the fixed-effects model (FEM) suggested a significant effect. Given the heterogeneity, REM is more appropriate, and the results do not support a consistent LDL-lowering effect of curcumin. Subgroup analyses suggested that study quality, regional differences, and outlier studies contributed to high heterogeneity.

**Conclusion:**

Curcumin effectively reduced levels of TG, TC, and BMI in patients with metabolic diseases. However, its effects on HDL-C and LDL-C were inconsistent and non-significant under random-effects models, indicating limited efficacy for these endpoints. Providing a more detailed context for the variability in lipid outcomes enhances understanding, especially for non-expert audiences.

## 1 Introduction

The global prevalence of metabolic diseases, including obesity, dyslipidemia, and insulin resistance, continues to rise, posing significant public health challenges and increasing the burden on healthcare systems worldwide (1–3). These conditions are major risk factors for cardiovascular diseases, type 2 diabetes, and other chronic health complications. Identifying effective interventions to manage metabolic dysfunction remains a critical area of research.

Curcumin, a bioactive compound derived from the spice turmeric, has garnered significant attention due to its wide-ranging therapeutic properties, including anti-inflammatory, antioxidant, and lipid-modifying effects (4–6). Beyond its traditional use in culinary and medicinal practices, curcumin has emerged as a promising candidate for managing metabolic parameters. Preclinical studies and randomized controlled trials (RCTs) have highlighted its potential in modulating blood lipid levels, body mass index (BMI), and other markers of metabolic health. For instance, Kiyama et al. (1) demonstrated curcumin’s capacity to regulate blood glucose and lipid levels in diabetic patients, while Gupta et al. (2) provided evidence of its efficacy in improving metabolic health outcomes.

Despite these promising findings, the therapeutic application of curcumin in metabolic diseases remains understudied and accompanied by inconsistencies. Variability in study outcomes is evident, particularly concerning blood lipid profiles and BMI, likely due to heterogeneity in study design, population characteristics, curcumin formulations, and dosages. Furthermore, while studies highlighted curcumin’s potential, a lack of synthesis and critical analysis of these findings limits the ability to draw robust conclusions about its clinical utility.

This gap reflects the need for a comprehensive meta-analysis to evaluate curcumin’s effects on key metabolic parameters, including blood lipids and BMI. By systematically examining the consistency, magnitude, and potential heterogeneity of curcumin’s effects, this study aimed to provide a clearer understanding of its role as a therapeutic agent in the management of metabolic diseases. The findings cannot only address existing gaps in the literature, but also provide valuable insights for guiding future research and clinical applications.

## 2 Methods

### 2.1 Search strategy

A comprehensive search of electronic databases including PubMed, Embase, Cochrane Central Register of Controlled Trials (CENTRAL), and Web of Science was conducted for the meta-analysis, targeting publications from January 2005 to September 2025. The search employed an extensive list of keywords and combinations to capture a broad spectrum of relevant studies. Key terms included “curcumin,” “turmeric,” “BMI,” “body mass index,” “blood lipids,” “lipid profile,” “type 2 diabetes,” “metabolic diseases,” “hyperlipidemia,” “randomized controlled trial,” and “RCT.” Boolean operators (AND, OR) were utilized to refine the search, enabling both specificity and sensitivity. Examples of combinations included “curcumin AND BMI,” “curcumin AND blood lipids,” “turmeric AND metabolic diseases,” and “RCTs in curcumin supplementation.”

### 2.2 Inclusion and exclusion criteria

The inclusion criteria required studies to be RCTs concentrating on the effects of curcumin or turmeric supplementation on BMI, blood lipids, or other related metabolic disease parameters. Only studies conducted on human participants were included, with full-text availability for thorough evaluation. Studies needed to report sufficient data on relevant outcomes, such as changes in BMI or lipid profiles, to be eligible for the meta-analysis.

The exclusion criteria excluded non-randomized studies, such as observational studies, case reports, and qualitative research. Studies conducted on animal models or through *in vitro* experiments were also excluded. Additionally, research not addressing the effects of curcumin or turmeric on BMI, blood lipids, or metabolic diseases, as well as studies without full-text availability, were ineligible. Reviews, meta-analyses, and other secondary analyses lacking original data were not considered. Studies with insufficient outcome data or sample sizes of fewer than 10 participants, which could compromise statistical reliability, were similarly excluded.

### 2.3 The risk of bias assessment

Cochrane RevMan (version 2.0) was used to assess the risk of bias. Begg’s test was used to evaluate publication bias through a rank correlation approach, while Egger’s test assessed bias using regression based on standard error. Funnel plots provided a visual assessment of publication bias and heterogeneity among studies.

### 2.4 Statistical analysis

The analysis primarily utilized the “meta” package in R (Version: 4.1.3), a comprehensive tool for conducting meta-analyses. Among the key statistical methods and visualizations, forest plots were employed to visually present individual study effects, their confidence intervals, and the overall meta-analysis result. The Baujat analysis identified studies exerting undue influence on the pooled effect size, guiding sensitivity analysis and ensuring the robustness of the results. Similarly, the Galbraith plot highlighted outlier studies and sources of heterogeneity, facilitating a deeper understanding of the variability among included studies. The quality of the included studies was rigorously evaluated using the Quality Assessment of Controlled Intervention Studies tool, as recommended by the National Heart, Lung, and Blood Institute (NHLBI).

The fixed-effects model (FEM) was used when heterogeneity, assessed through I^2^ and related statistics, was minimal, assuming that all studies estimated the same underlying effect size. In contrast, the random-effects model (REM) was applied to account for between-study variance when significant heterogeneity was detected. The combined use of both models provides a robust interpretation of the pooled effects, accommodating variations across studies and ensuring the validity of the findings under different assumptions. To explore the sources of heterogeneity, subgroup analysis and meta-regressions were conducted, concentrating on study-level characteristics, such as intervention dosage, duration, and participant demographics (e.g., baseline BMI or lipid levels). For instance, higher doses of curcumin supplementation were associated with more consistent improvements in lipid profiles, highlighting potential dose-dependent effects. Additionally, differences in study designs and target populations contributed to the observed variability in outcomes. Data extraction was performed independently by two reviewers using a standardized form. Discrepancies were resolved through discussion or by consulting a third reviewer. Extracted data included study characteristics, participant demographics, intervention details, and outcome measures.

## 3 Results

### 3.1 Characteristics and quality of the included studies in the meta-analysis of curcumin effects on blood lipids and BMI

**Figure 1:**
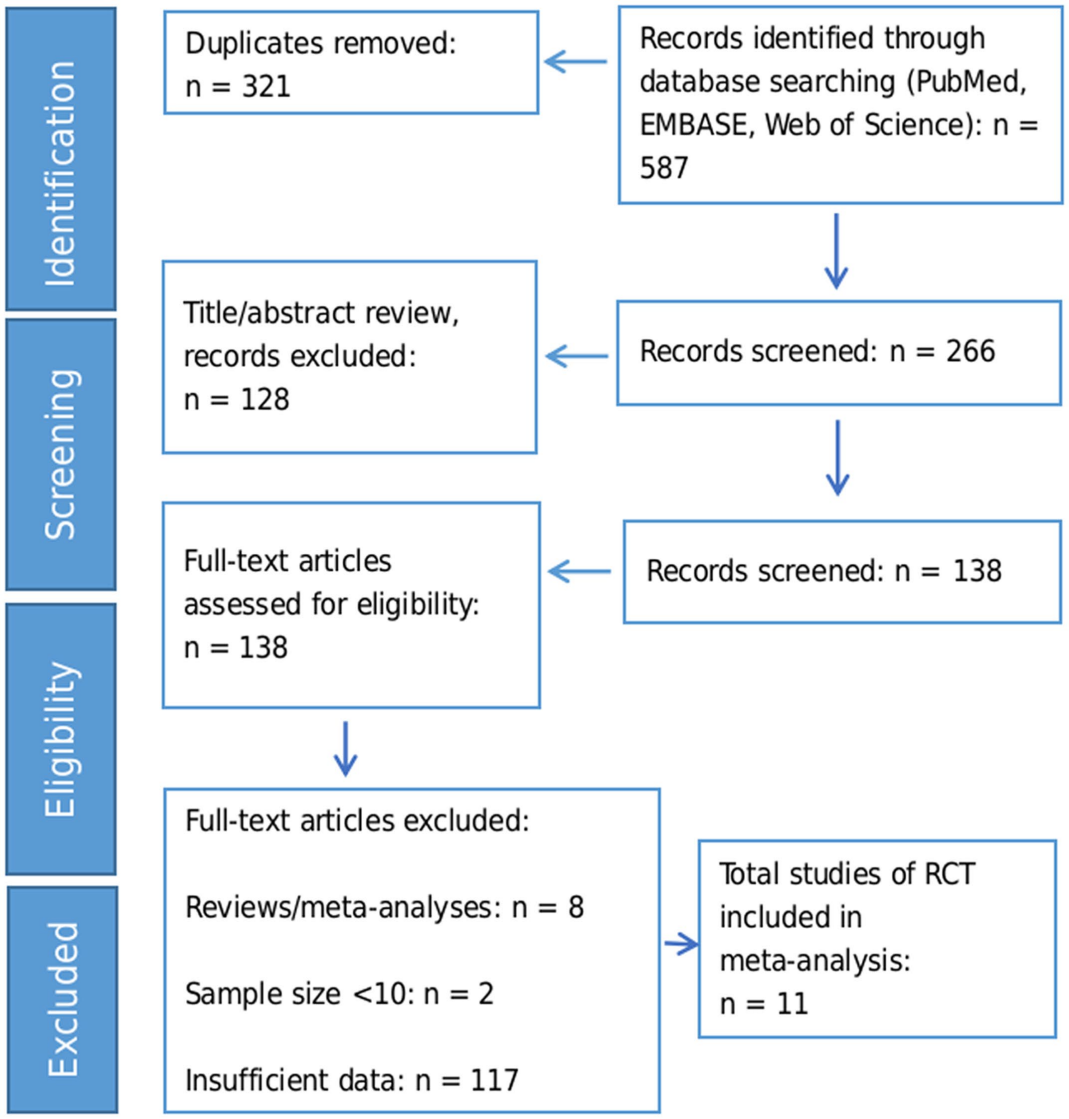
Flow diagram of study selection process.

We identified 587 records through database searching. After removing 321 duplicates, 266 records remained for title/abstract screening. Of these, 128 records were excluded based on title/abstract criteria, leaving 138 full-text articles for detailed assessment. Finally, 11 RCTs with a total of 662 participants were included in the meta-analysis. The flow diagram of the study selection process is exhibited in Figure 1.

The studies were conducted in four countries: Iran (9–16), China (17), India (18), and Australia (19). The majority of the studies (9 out of 11) were from Iran, and they had a fair or good quality rating. The study by Na et al. (3) was from China, and it had a good quality rating (Table 1). The study by Usharani et al. (4) was from India, and it had a fair quality rating. The study by Thota et al. (5) was from Australia, and it had a fair quality rating. The studies varied in their study design, RCT ID, sample size, age, and gender. The details of each study are shown in the Table 2.

**Table 1:** Summary of included literatures.

Study ID (PMID)	Country	Status	Included or excluded	Summary/Title	Ref.
PMID: 30859660 (IRCT201204162602)	Iran	Completed	Included	A double-blind, randomized clinical trial that examines the efficacy of turmeric supplementation on glycemic status, lipid profile, hs-CRP, and total antioxidant capacity in hyperlipidemic T2D patients.	Adab et al. (6)
PMID: 30864188 (NCT02529969)	Iran	Completed	Included	A randomized, double-blind, placebo-controlled trial that examines the effects of curcumin supplementation on high-sensitivity C-reactive protein, serum adiponectin, and lipid profile in T2D patients.	Adibian et al. (17)
PMID: 27761427 (IRCT2013081114330 N1)	Iran	Completed	Included	A double-blind, randomized clinical trial that evaluated the efficacy of nano-curcumin on HbA1c, fasting blood glucose, and lipid profile in T2D patients.	Rahimi et al. (18)
PMID: 38020740 (IRCT20131009014957 N4)	Iran	Completed	Included	A triple-blind randomization clinical trial that investigates the effect of *Nigella sativa* (NS), curcumin nanomicelle (CN), lipid profile, glycemic status and 17- β estradiol (ES) levels in postmenopausal women.	Sadeghzadeh et al. (8)
PMID: 36192751 (IRCT20180201038585 N2)	Iran	Completed	Included	A randomized, double-blind, placebo-controlled, 2×2 factorial trial that assessed the effects of curcumin and/or coenzyme Q10 supplementation on metabolic syndrome components including systolic blood pressure (SBP), diastolic blood pressure (DBP), waist circumference (WC), triglyceride (TG), and high density.	Sangouni et al. (7)
PMID: 35090529 (IRCT20190902044671 N1)	Iran	Completed	Included	A phase 2 randomized, multi-arm, parallel-group, double-blind placebo-controlled clinical trial that investigates the effect of curcumin and zin co-supplementation along with a loss-weight diet on lipid profiles in patients with prediabetes.	Karandish et al. (9)
PMID: 32655024 (IRCT20190902044671 N1)	Iran	Completed	Included	A randomized, double-blind, placebo-controlled trial that assessed the effects of nano-curcumin on metabolic status in patients with diabetes on haemodialysis.	Shafabakhsh et al. (32)
PMID: 28735818 (IRCT201505301165 N4)	Croatia, Iran, United States	Completed	Included	A randomized controlled trial assessing whether the combination of curcuminoids and piperine reduce the serum lipid in patients with T2D.	Panahi et al. (10)
PMID: 22930403 (ISRCTN85826075)	China	Completed	Included	A double-blind, placebo-controlled trial that investigates the glucose-lowering effect of curcuminoids in patients with type 2 diabetes (T2D).	Na et al. (3)
PMID: 18588355	India	Completed	Included	A randomized, parallel-group, placebo-controlled, 8-week study that investigates the effect of NCB-02, atorvastatin, and placebo on endothelial function, oxidative stress, as well as inflammatory markers in patients with type 2 diabetes mellitus (T2DM).	Usharani et al. (4)
PMID: 30684965 (ACTRN12615000559516)	Australia, New Zealand	Completed	Included	Curcumin and/or omega-3 polyunsaturated fatty acids supplementation reduces insulin resistance and blood Lipids in individuals with high risk of type 2 diabetes: a randomised controlled trial.	Thota et al. (5)

**Table 2:** All data extracted from primary search.

Study ID	Region/Country	S.D.	Sample size (n)			Age		Gender (no., %)				TG levels (mmol. L) [Mean ± SD, median (IQR)]				TC level (mmol/L, %) (Mean ± SD)				HDL-C levels (Mean ± SD)				LDL-C levels (Mean ± SD)				BMI (kg/m^2^) (Mean ± SD)				Ref.
			Total (n)	Curc. (n)	Plac. (n)	Curc.	Plac.	M		F		Curc.		Plac.		Curc.		Plac.		Curc.		Plac.		Curc.		Plac.		Curc.		Plac.		
								Curc.	Plac.	Curc.	Plac.	BL	P.I.	BL	P.I.	BL	P.I.	BL	P.I.	BL	P.I.	BL	P.I.	BL	P.I.	BL	P.I.	BL	P.I.	BL	P.I.	
N/A	India	RCT	72	NA	NA	NA	NA	NA	NA	NA	NA	NA	NA	NA	NA	NA	NA	NA	NA	NA	NA	NA	NA	NA	NA	NA	NA	NA	NA	NA	NA	Rochlani et al. (33)
ISRCTN 85,826,075	China	RCT	100	50	50	55.42 ± 6.40	54.72 ± 8.34	24 (48)	25 (50)	NA	NA	2.23 ± 0.53	1.78 ± 0.56	2.19 ± 1.04	2.11 ± 0.75	6.11 ± 1.13	5.58 ± 1.08	6.08 ± 1.24	5.90 ± 1.19	1.37 ± 0.26	1.42 ± 0.29	1.33 ± 0.28	1.34 ± 0.23	4.30 ± 1.20	3.80 ± 1.03	4.32 ± 1.15	4.15 ± 1.17	27.12 ± 3.04	NA	27.42 ± 3.04	NA	Fahed et al. (34)
IRCT201 30,811,143 30 N1	Iran	RCT	70	35	35	56.34 ± 11.17	60.95 ± 10.77	17 (48.5)	14 (40)	18 (51.5)	21 (60)	109 (94.75)	142 (97.50)	131 (60.27)	113 (58.0)	163.4 ± 33.94	162.4 ± 38.59	158.62 ± 44.06	149.00 ± 24.62	54.30 ± 14.02	60.35 ± 15.96	60.95 ± 15.68	55.00 ± 11.09	96.57 ± 33.94	99.78 ± 30.33	91.04 ± 28.72	84.00 ± 12.59	26.92 ± 2.71	27.27 ± 3.59	25.57 ± 2.71	27.50 ± 3.38	Chew et al. (30)
IRCT2015 053011 65 N4	Croatia, Iran, U.S.	RCT	100	50	50	43 ± 8	41 ± 7	25	26	25	24	229.78 ± 81.84	205.48 ± 64.52	207.62 ± 54.63	187.06 ± 44.34	217.34 ± 41.60	195.48 ± 33.39	231.04 ± 70.95	213.98 ± 55.12	40.86 ± 5.41	42.42 ± 4.33	39.46 ± 6.09	39.24 ± 5.93	169.16 ± 30.77	160.94 ± 28.32	199.06 ± 54.49	168.90 ± 29.91	26.53 ± 2.32	26.04 ± 2.35	27.33 ± 1.58	27.57 ± 1.63	Salehi et al. (35)
ACTRN1 2,615,000 559,516	Australia, New Zealand	RCT	64	15	16	55 ± 2.8	50 ± 2.5	6	7	9	9	NA	NA	NA	NA	NA	NA	NA	NA	NA	NA	NA	NA	NA	NA	NA	NA	30.9 ± 1.2	NA	31.9 ± 1.7	NA	Zeng et al. (36)
IRCT201 204,162,602	Iran	RCT	75	39	36	54.76 ± 6.00	55.66 ± 8.64	19 (48.7)	17 (47.2)	20 (51.0)	19 (52.8)	181.56 ± 79.79	141.74 ± 52.02	164.05 ± 81.19	197.05 ± 96.98	148.85 ± 36.11	149.82 ± 35.67	155.36 ± 36.27	176.88 ± 37.58	38.79 ± 10.30	37.07 ± 9.12	44.63 ± 10.66	42.11 ± 9.39	82.56 ± 20.99	75.23 ± 18.84	86.61 ± 21.99	89.05 ± 21.46	28.98 ± 3.68	28.26 ± 3.45	28.82 ± 4.96	28.68 ± 4.86	Jiang et al. (37)
NCT025 29,969	Iran	RCT	53	21	23	58 ± NA	60 ± 7	13 (61.6)	9 (39.1)	8 (38.4)	14 (60.9)	124 ± 36	109 ± 36	126 ± 52	121 ± 44	167 ± 34	163 ± 39	180 ± 47	175 ± 47	30 ± 2	30 ± 2	30 ± 2	32 ± 5	112 ± 31	108 ± 36	125 ± 44	118 ± 47	NA	NA	NA	NA	Kiyama (1)
IRCT201 50,606,022 562 N6	Iran	RCT	60	30	30	NA	NA	NA	NA	NA	NA	NA	NA	NA	NA	NA	NA	NA	NA	NA	NA	NA	NA	NA	NA	NA	NA	NA	NA	NA	NA	Gupta et al. (2)
IRCT201 90,902,044 671 N1	Iran	RCT	82	21	20	36.95 ± 7.23	34.19 ± 7.03	NA	NA	16 (76.2)	12 (60)	131.48 ± 26.16	107.48 ± 11.9	132.55 ± 25.96	123.1 ± 18.6	188.95 ± 23.5	175.71 ± 17.01	186.5 ± 24.91	184.25 ± 24.4	47 ± 6.39	56.47 ± 6.14	48.25 ± 5.58	50.15 ± 5.34	114.63 ± 20.22	97.74 ± 18.9	111.74 ± 22.29	109.48 ± 22.77	30.97 ± 2.33	NA	30.46 ± 2.75	NA	Adab et al. (6)
IRCT2018 0201038 585 N2	Iran	RCT	88	22	22	38.8 ± 4.9		14 (64)		8 (36)		224.7 ± 66.9	166.7 ± 72.8	224.7 ± 66.9	166.7 ± 72.8	217.4 ± 37.4	186.5 ± 30.4	217.4 ± 37.4	186.5 ± 30.4	32.0 ± 5.6	42.0 ± 5.9	32.0 ± 5.6	42.0 ± 5.9	89.4 ± 6.5	75.3 ± 5.4	89.4 ± 6.5	75.3 ± 5.4	30.0 ± 4.6	29.4 ± 4.2	30.0 ± 4.6	29.4 ± 4.2	Adibian et al. (17)
IRCT201 31,009,014 957 N4	Iran	RCT	120	30	30	58.0 (3.4)	58.4 (3.4)	NA	NA	NA	NA	116.7 ± 26.87	108.66 ± 18.81	116.6 ± 25.7	102.5 ± 19.0	208.17 ± 45.01	166.44 (30.41)	183.51 (55.41)	171.50 (32.23)	43.88 ± 5.84	47.60 ± 9.39	42.23 ± 6.45	47.40 ± 11.23	141.05 ± 48.45	137.33 ± 43.88	117.94 ± 58.42	112.77 ± 55.03	27.7 ± 3.8	NA	28.8 ± 3.8	NA	Rahimi et al. (18)

*S.D., study design; Curc., curcumin; Plac., placebo; BL, baseline; P.I., post-intervention; RCT, randomized controlled trial; Ref., reference; BMI, body mass index; TG, triglyceride; TC, total cholesterol; HDL-C, high density lipoprotein - cholesterol; LDL-C, low density lipoprotein – cholesterol; M, male; F, Female.

### 3.2 The effects of curcumin on TG levels

**Figure 2:**
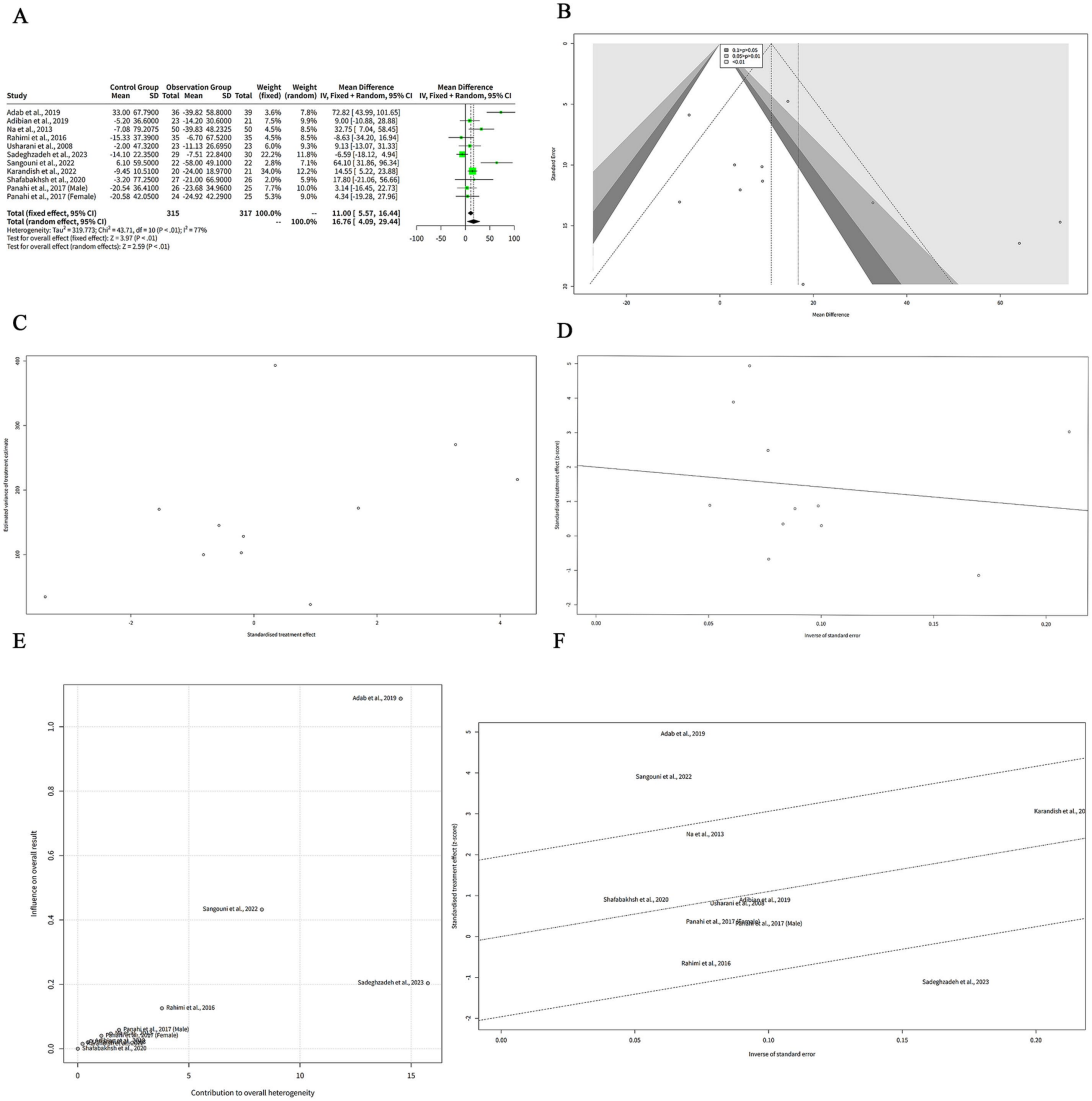
The effect of curcumin on TG levels. **(A)** Forest plot of the MDs in TG levels between curcumin and control groups. **(B)** Funnel plot of the MDs in TG levels. **(C)** Rank correlation test of funnel plot asymmetry using Begg method. **(D)** Linear regression test of funnel plot asymmetry using Egger method. **(E)** Baujat plot of the contribution and influence of each study on the overall effect and heterogeneity. **(F)** Galbraith plot of the standardized effect estimates.

We extracted the mean and SD of TG levels at baseline and post-intervention for each group (Table 2). The FEM showed that the MD between the curcumin and control groups was 11.00 mg/dL, which suggests that curcumin intervention was associated with a greater reduction in TG levels compared to the control group. The 95% CI of the MD was (−16.44, −5.57), which means that we are 95% confident that the true MD lies between −16.44 and −5.57 mg/dL. The z-value of the MD was −3.97, which means that the MD was 3.97 standard errors away from zero. The *p*-value of the MD was < 0.001, which means that the probability of observing such a large MD by chance alone was less than 0.1%. The REM showed a greater reduction in TG levels with curcumin supplementation (MD: −16.76 mg/dL). The 95% CI of the MD was (−29.44, −4.09), which means that we are 95% confident that the true MD lies between −29.44 and −4.09 mg/dL. The z-value of the MD was −2.59, which means that the MD was 2.59 standard errors away from zero. The *p*-value of the MD was 0.010, which means that the probability of observing such a large MD by chance alone was 1%. The negative MD indicates that curcumin was associated with a significant reduction in TG levels compared to control (Figure 2A).

The REM showed a larger effect size than the FEM, but also a wider confidence interval. The heterogeneity among the studies was high, as indicated by the *Q* statistic (*Q* = 43.71, df = 10, *p* < 0.001), the I^2^ statistic (I^2^ = 77, 95% CI: 59–87%), and the H statistic (*H* = 2.09, 95% CI: 1.56–2.79).

Figure 2B shows a funnel plot of the effects of curcumin on TG levels in patients with metabolic diseases. The funnel plot suggesting that there is some degree of publication bias or heterogeneity among the studies. Most of the studies are clustered in the upper middle part of the plot, indicating that they have high precision and small effect sizes. Two studies, Adab et al. (6) and Sangouni et al. (7), are outliers in the lower right part of the plot, indicating that they have low precision and large effect sizes. There are no studies in the lower left part of the plot, indicating that there is a lack of studies with low precision and negative effect sizes. This may imply that some studies with unfavorable results have not been published or included in the meta-analysis.

We performed two statistical tests to assess the funnel plot asymmetry in our meta-analysis of the effects of curcumin on TG levels in patients with metabolic diseases. The rank correlation test is based on the Kendall rank correlation coefficient (ks) between the effect estimates and their variances. A significant correlation suggests that the effect estimates are related to their precision, which may indicate funnel plot asymmetry. The test result showed that the ks was 21.00, with a standard error of 12.85. The *z*-value was 1.63, and the *p*-value was 0.10. This means that the correlation was not statistically significant at the 5% level, and we could not reject the null hypothesis of no funnel plot asymmetry (Figure 2C). The linear regression test is based on the weighted linear regression of the effect estimates on their standard errors. The slope of the regression line (bias) represents the degree of funnel plot asymmetry. A significant slope suggests that the effect estimates are influenced by their standard errors, which may indicate funnel plot asymmetry. The test result showed that the bias was 2.00, with a standard error of 1.41. The intercept was −5.63, with a standard error of 12.99. The t-value was 1.41, with 9 degrees of freedom, and the *p*-value was 0.19. This means that the slope was not statistically significant at the 5% level, and we could not reject the null hypothesis of no funnel plot asymmetry (Figure 2D). Both tests did not provide evidence of funnel plot asymmetry in our meta-analysis. However, these tests have low power and may fail to detect asymmetry when it exists.

The Baujat analysis was conducted to assess sources of heterogeneity in the meta-analysis of curcumin’s effect on TG levels in patients with metabolic diseases. The Baujat plot in Figure 2E demonstrated that most studies were clustered in the lower-left quadrant, indicating low contribution and low influence. This suggests that these studies align with the overall effect and contribute minimally to heterogeneity. In contrast, few outliers were identified. Adab et al. (6) appeared in the upper-right quadrant, reflecting high contribution and high influence, signifying a substantial deviation from the overall effect and a significant contribution to heterogeneity. Additionally, Sangouni et al. (7) and Sadeghzadeh et al. (8) were positioned in the upper region of the plot, indicating low contribution, while high influence. Although these studies did not markedly contribute to heterogeneity, they exerted significant impact on the pooled effect.

The Galbraith analysis was conducted to investigate the sources of heterogeneity in the effect of curcumin on TG levels in patients with metabolic diseases. The Galbraith plot in Figure 2F revealed the distribution of studies across four regions corresponding to the plot’s quadrants. Adab et al. (6) and Sangouni et al. (7) were positioned in the upper-right region, indicating large positive effect estimates and large standard errors. These studies report that curcumin was associated with a significant reduction in TG levels compared to control, while they have low precision and high uncertainty. Sadeghzadeh et al. (8) appeared in the lower-right region, reflecting a large negative effect estimate and a large standard error. This study reported that curcumin increased TG levels compared to the control, while exhibited low precision and high uncertainty. The remaining studies were located in the lower-left and upper-left regions, representing small effect estimates and small standard errors. Although Begg’s and Egger’s tests did not indicate significant funnel plot asymmetry, their statistical power is limited due to the small number of studies (*n* < 10). This low power may reduce the reliability of these tests in detecting potential publication bias or heterogeneity.

### 3.3 Subgroup meta-analysis of the effects of curcumin on TG levels

**Figure 3:**
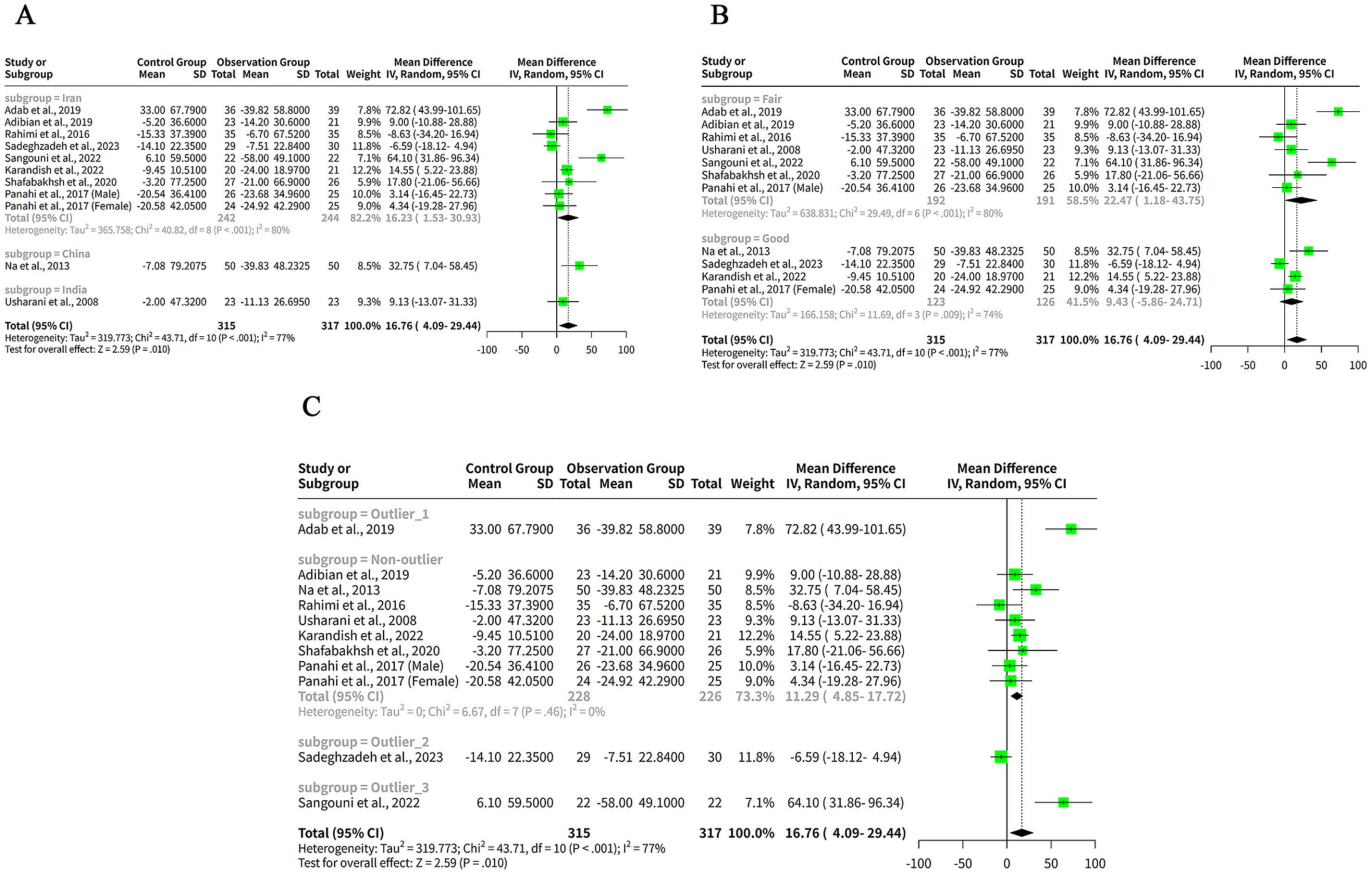
The effect of curcumin on TG levels by subgroup analysis. **(A)** Forest plot of the subgroup analysis based on the region or country of the studies. **(B)** Forest plot of the subgroup analysis based on the quality assessment of the studies. **(C)** Forest plot of the subgroup analysis based on the outlier status of the studies.

To investigate the reasons behind the high heterogeneity observed in the meta-analysis of curcumin’s effect on TG levels in patients with metabolic diseases, subgroup analysis was performed based on the region or country of the studies. The results indicated that the MDs were positive and significant for studies conducted in Iran and China, suggesting that curcumin effectively reduced TG levels compared to the control in these regions. In contrast, the MD for India was positive, while not significant, demonstrating no notable effect of curcumin on TG levels in this region. Substantial heterogeneity was detected in the Iran subgroup (I^2^ = 80%), reflecting significant variation among the studies. Heterogeneity in the China and India subgroups was not assessed, as each subgroup included only one study. The heterogeneity between the subgroups was also high (*Q* = 43.71, df = 10, *p* < 0.001) (Figure 3A), indicating that there was significant difference in the effect sizes across the subgroups. Thus, the subgroup analysis revealed that the region or country of the studies was a potential source of heterogeneity in our meta-analysis, while it may not necessarily be the primary cause of heterogeneity (Figure 3A).

To explore the reasons for the high heterogeneity in our meta-analysis of the effects of curcumin on TG levels in patients with metabolic diseases, we conducted a subgroup analysis based on the quality assessment of the studies. The subgroup analysis showed that the MDs were positive for both subgroups, indicating that curcumin reduced TG levels compared to control in both quality categories. However, the MD was only significant for the Fair subgroup, indicating that curcumin had a larger and more consistent effect on TG levels in studies with lower quality. The MD for the Good subgroup was not significant, indicating that curcumin had a smaller and more variable effect on TG levels in studies with higher quality. The heterogeneity within both subgroups was high (I^2^ = 80% for Fair and I^2^ = 74% for Good) (Figure 3B), indicating that there was substantial variation among the studies in each subgroup. The heterogeneity between the subgroups was also high (Q = 43.71, df = 10, *p* < 0.001) (Figure 3B), indicating that there was significant difference in the effect sizes across the subgroups. Thus, the subgroup analysis revealed that the quality assessment of the studies was a potential source of heterogeneity in our meta-analysis, while it may not necessarily be the primary cause of heterogeneity (Figure 3B).

To explore the reasons for the high heterogeneity in our meta-analysis of the effects of curcumin on TG levels in patients with metabolic diseases, we conducted a subgroup analysis based on the outlier status of the studies. The non-outlier subgroup has eight studies, with a small and significant positive MDs of 11.29 mg/dL and a narrow confidence interval of (4.85, 17.72) mg/dL (Figure 3C). The tau^2^ and tau values are zero for this subgroup, indicating no heterogeneity among the studies. The Q value is 6.67 (Figure 3C), indicating no significant heterogeneity. The I^2^ value is 0%, indicating no heterogeneity (Figure 3C).

### 3.4 The effects of curcumin on TC levels

**Figure 4:**
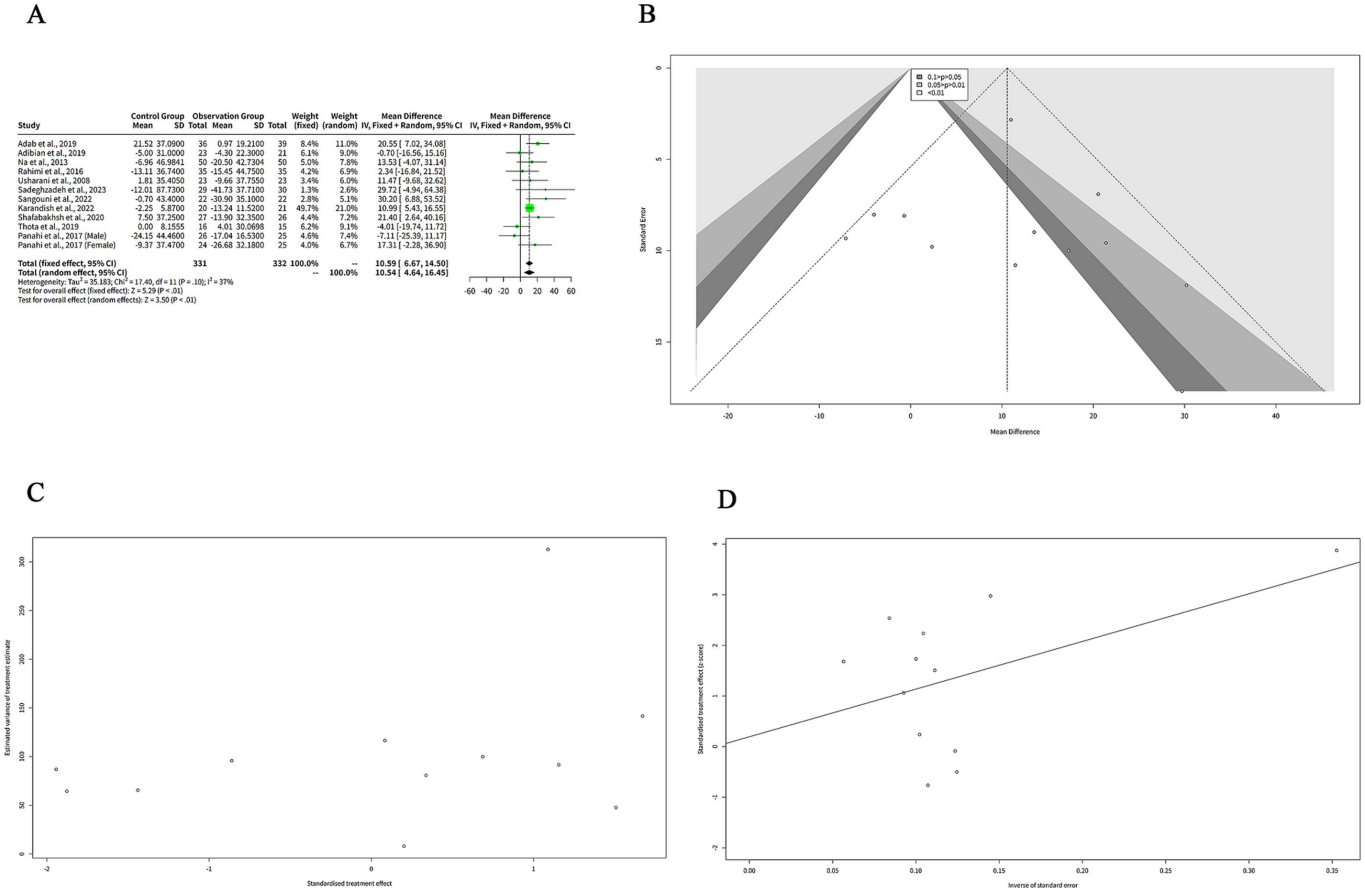
The effect of curcumin on TC levels. **(A)** Forest plot of the MDs in TC levels between curcumin and control groups. **(B)** Funnel plot of the MDs in TC levels. **(C)** Rank correlation test of funnel plot asymmetry using Begg method. **(D)** Linear regression test of funnel plot asymmetry using Egger method.

The results showed that curcumin had a significantly great reduction in TC levels compared to placebo in both models, with a MD of 10.59 mg/dL (95%-CI: 6.67–14.50, z = 5.29, *p* < 0.001) in the FEM and a MD of 10.54 mg/dL (95%-CI: 4.64–16.45, z = 3.50, *p* < 0.001) in the REM (Figure 4A). The confidence intervals of the MDs were narrow, indicating a high precision of the estimates. The heterogeneity among the studies was moderate, with a tau^2^ of 35.18, a tau of 5.93, an I^2^ of 37%, an H of 1.26, and a Q of 17.40 (df = 11, *p* = 0.10) (Figure 4A). These statistics suggest that there was some variation in the effect sizes across the studies, while not enough to invalidate the meta-analysis. The choice of the model did not affect the results substantially, as the MDs and the confidence intervals were very similar in both models. However, the FEM may be more suitable for this analysis, as the I^2^ of 37% indicates low heterogeneity among the studies and provides a more precise estimate of the MD (Figure 4A).

The funnel plot in Figure 4B is relatively symmetrical, with most studies concentrated in the middle and lower part of the graph. This suggests that there is no significant publication bias or heterogeneity among the studies. However, one study by Sadeghzadeh et al. (8) is located at the bottom right corner of the plot, indicating a large and positive effect size with low precision. This study is an outlier and may have a different population or intervention than the other studies.

We performed two statistical tests (Begg and Egger) to assess the funnel plot asymmetry in Figures 4C,D, respectively, which could indicate the presence of publication bias or other sources of heterogeneity in the meta-analysis. Figure 4C shows the result of the rank correlation test. The test result was *z* = 1.10, with a *p*-value of 0.27. The sample estimates were ks = 16.00 and se.ks = 14.58. The null hypothesis of this test is that there is no correlation between the effect size and its variance, which means that the funnel plot is symmetrical. The alternative hypothesis is that there is a correlation, which means that the funnel plot is asymmetrical. Since the *p*-value was greater than 0.05, we failed to reject the null hypothesis and concluded that there was no evidence of funnel plot asymmetry based on the rank correlation test.

Figure 4D shows the result of the linear regression test. The test result was t = 0.25, with 10 degrees of freedom and a *p*-value of 0.80. The sample estimates were bias = 0.19, se.bias = 0.76, intercept = 9.42, and se.intercept = 5.29. The details of the test were that the multiplicative residual heterogeneity variance (tau^2^) was 1.73, the predictor was the standard error, and the weight was the inverse variance. The null hypothesis of this test is that the intercept term is zero, which means that the funnel plot is symmetrical.

### 3.5 The effects of curcumin on HDL-C levels

**Figure 5:**
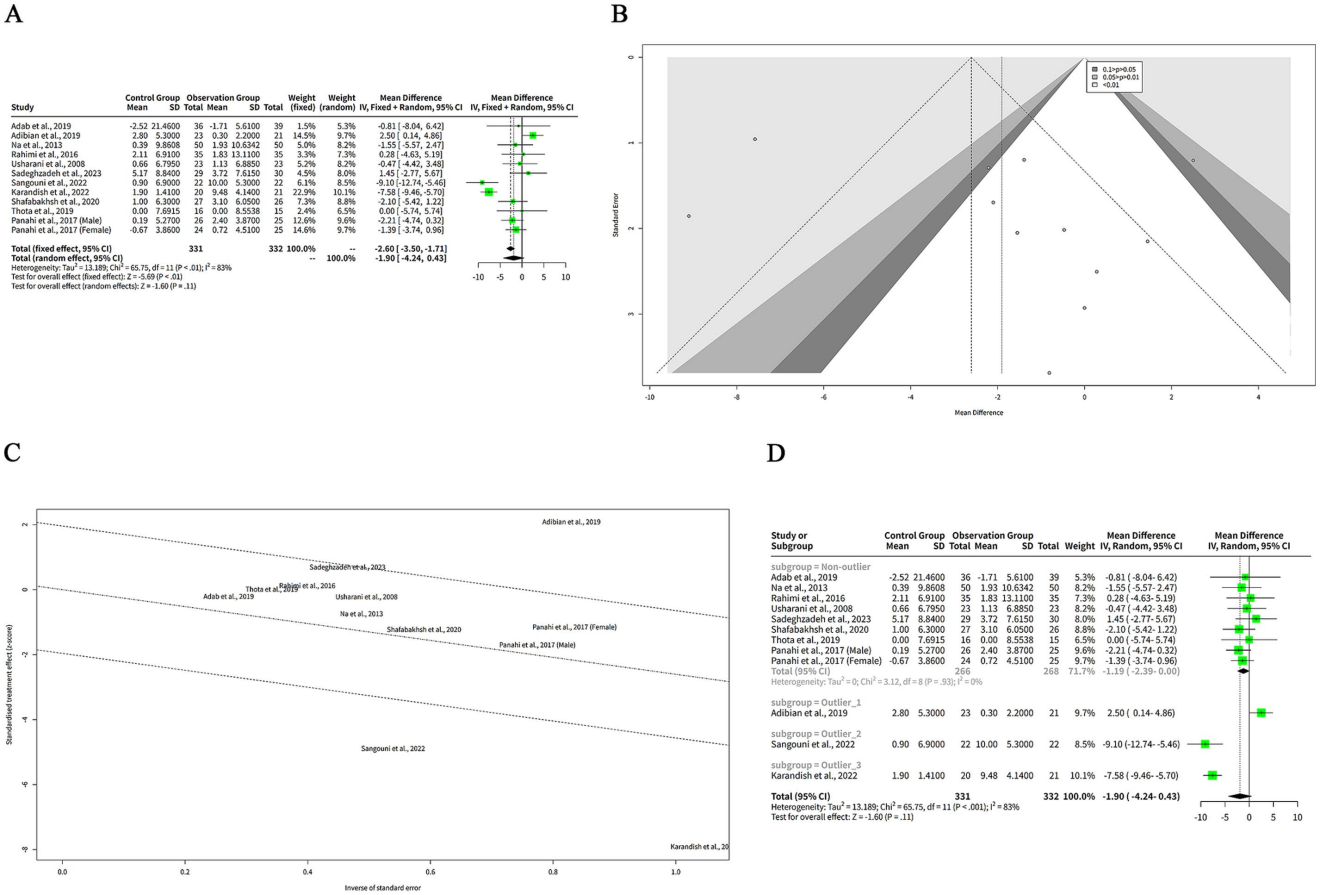
The effect of curcumin on HDL-C levels. **(A)** Forest plot of the effect of curcumin on HDL-C levels. **(B)** Funnel plot of the effect of curcumin on HDL-C levels. **(C)** Galbraith plot of the effect of curcumin on HDL-C levels. **(D)** Forest plot of the subgroup analysis of the effect of curcumin on HDL-C levels.

We extracted the mean SD of HDL-C levels from each study and pooled them using both FEM and REM. Our findings indicated that curcumin intervention led to a significantly greater elevation in HDL-C levels compared to placebo when analyzed with the FEM, with a MD of −2.60 mg/dL (95% CI: −3.50 to −1.71, *z* = −5.69, *p* < 0.001) (Figure 5A). Despite the significant results from the FEM, the REM analysis showed no significant difference, with a MD of −1.90 mg/dL (95% CI: −4.24 to 0.43, *z* = −1.60, *p* = 0.11) (Figure 5A), reflecting the variability among the included studies. The heterogeneity among studies was high, with a tau^2^ of 13.19 (95%-CI: 3.42–37.16), a tau of 3.63 (95%-CI: 1.85–6.10), an I^2^ of 83% (95%-CI: 72–90%), and an H of 2.44 (95%-CI: 1.90–3.15) (Figure 5A). The Q statistic was 65.75 with 11 degrees of freedom and *p* < 0.001, indicating that the heterogeneity was statistically significant (Figure 5A).

The funnel plot in Figure 5B appears relatively symmetrical, with most studies clustered in the middle and lower sections of the graph, suggesting minimal publication bias or heterogeneity among the studies. However, one study by Adab et al. (6) is positioned at the bottom of the plot, reflecting a large negative effect size with low precision. This outlier may differ from other studies in terms of population characteristics or intervention protocols.

Another plot used to evaluate the heterogeneity of the studies was the Galbraith plot, as illustrated in Figure 5C. From the Galbraith plot, it is evident that all included studies were distributed across the four regions. One study by Adab et al. (6) was located in the first region from the top, indicating a large negative effect size with low precision. This study is an outlier and might influence the overall result. Two studies by Sangouni et al. (7) and Karandish et al. (9) were positioned in the fourth region from the top, suggesting large positive effect sizes with low precision. These studies were also outliers and could contribute to the observed heterogeneity. The remaining studies fell in the second and third regions, indicating moderate effect sizes with moderate-to-high precision. These studies were more consistent and aligned closely with the pooled effect size.

To explore the reasons for the high heterogeneity of the studies, we performed a subgroup analysis based on the outlier or non-outlier classification from the Galbraith plot. The results of the subgroup analysis are shown in the table below. The non-outlier subgroup consisted of nine studies. The MD of this subgroup was −1.19 mg/dL (95%-CI: −2.39-0.00) (Figure 5D), which was not statistically significant. The heterogeneity of this subgroup was low, with a tau^2^ of 0, a tau of 0, a Q of 3.12, and an I^2^ of 0% (Figure 5D). This suggests that the non-outlier studies were consistent and homogeneous (Figure 5D). The discrepancy between FEM and REM results for HDL-C underscores the influence of between-study heterogeneity. Caution is warranted in interpreting these findings, and the REM results should be prioritized given the high variability.

### 3.6 The effects of curcumin on LDL-C levels

**Figure 6:**
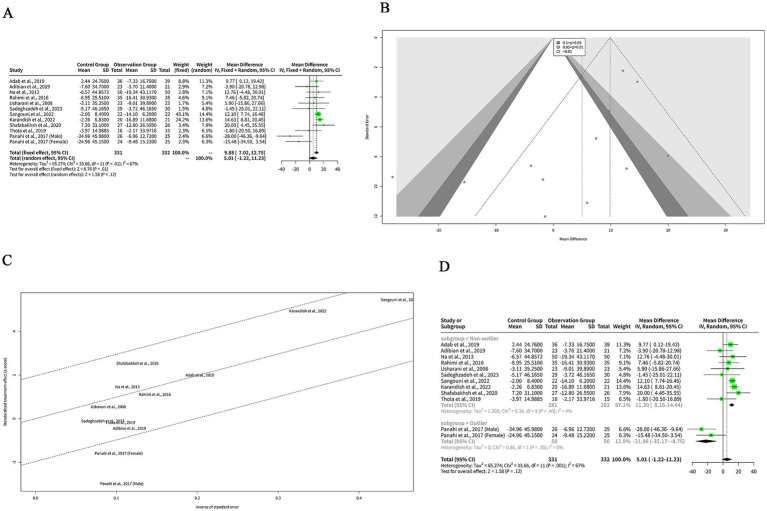
The effect of curcumin on LDL-C levels. **(A)** Forest plot of the effect of curcumin on LDL-C levels. **(B)** Funnel plot of the effect of curcumin on LDL-C levels. **(C)** Galbraith plot of the effect of curcumin on LDL-C levels. **(D)** Forest plot of the subgroup analysis of the effect of curcumin on LDL-C levels.

We extracted the mean SD of LDL-C levels from each study and pooled them using both FEM and REM. The analysis revealed that curcumin led to a more pronounced reduction in LDL-C levels compared to placebo when assessed with the FEM, indicating a MD of 9.88 mg/dL (95% CI: 7.02–12.75, z = 6.76, *p* < 0.001) (Figure 6A), signifying that curcumin is notably effective in lowering LDL-C. In contrast, under the REM, the reduction in LDL-C levels by curcumin was not statistically significant, with an MD of 5.01 mg/dL (95% CI: −1.22 to 11.23, *z* = 1.58, *p* = 0.12) (Figure 6A), suggesting that when accounting for variability among studies, the effect of curcumin on LDL-C levels is not conclusively different from placebo. The heterogeneity among studies was high, with a tau^2^ of 65.27 (95%-CI: 26.47–423.96), a tau of 8.08 (95%-CI: 5.15–20.59), an I^2^ of 67% (95%-CI: 40–82%), and an H of 1.75 (95%-CI: 1.29–2.37) (Figure 6A). The Q statistic was 33.66 with 11 degrees of freedom and *p* < 0.001, indicating that the heterogeneity was statistically significant (Figure 6A). The funnel plot in Figure 6B is relatively symmetrical, with most studies concentrated in the middle and lower part of the graph. This suggests that there is no significant publication bias or heterogeneity among the studies.

The Galbraith plot of our meta-analysis is shown in Figure 6C. We observed that all the included studies were distributed in the four regions. The studies of Panahi et al. (10) (Male subgroup) and Panahi et al. (10) (Female subgroup) were in the fourth region from the top, which means that they had large and positive effect sizes with low precision. These studies were outliers and may have introduced heterogeneity. The other studies were in the second and third regions, which means that they had moderate effect sizes with moderate or high precision.

To explore the reasons for the high heterogeneity of the studies in our meta-analysis of the effects of curcumin on LDL-C levels in patients with metabolic diseases, we performed a subgroup analysis based on the outlier or non-outlier classification from the Galbraith plot (Figure 6D). The non-outlier subgroup consisted of 10 studies with a total of 593 participants. The MD of this subgroup was 11.30 mg/dL (95%-CI: 8.16–14.44) (Figure 6D), which was statistically significant. The heterogeneity of this subgroup was low, with a tau^2^ of 1.209, a tau of 1.0995, a Q of 9.39, and an I^2^ of 4% (Figure 6D). This suggests that the non-outlier studies were consistent and homogeneous.

### 3.7 The effects of curcumin on BMI

**Figure 7:**
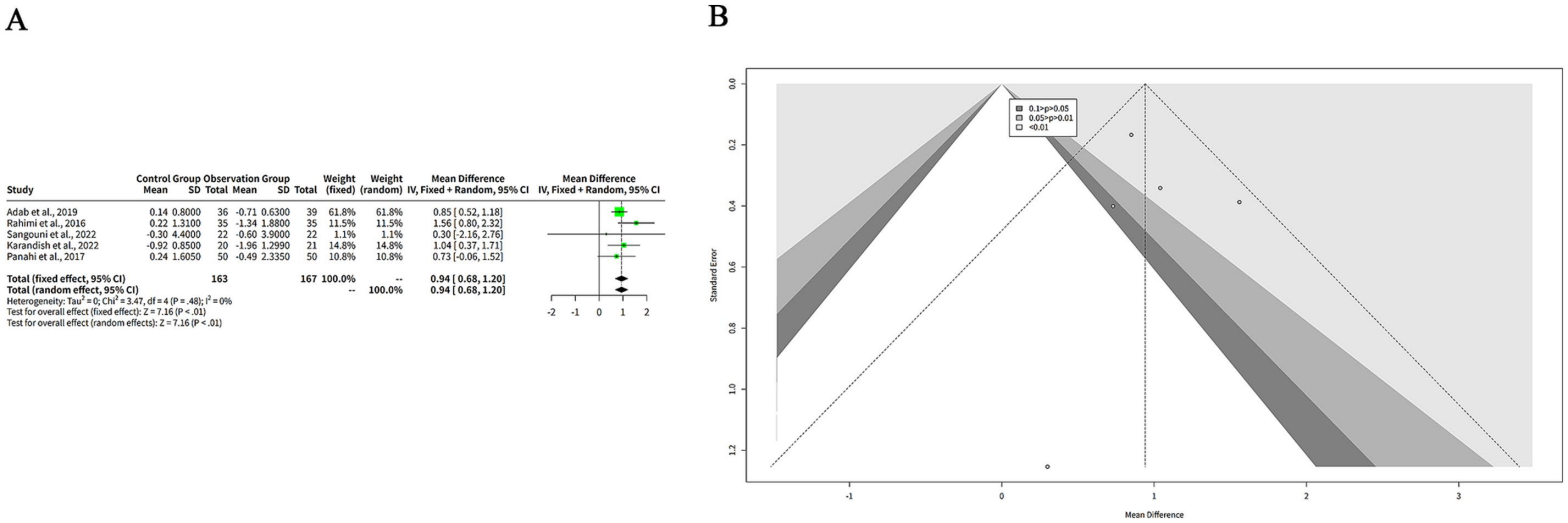
The effect of curcumin on BMI. **(A)** Forest plot of the effect of curcumin on BMI. **(B)** Funnel plot of the effect of curcumin on BMI.

We extracted the mean and SD of BMI from each study and pooled them using both FEM and REM. The results showed that curcumin had a significantly greater reduction on BMI compared to placebo in both models, with a MD of 0.94 kg/m^2^ (95%-CI: 0.68–1.20, *z* = 7.16, *p* < 0.001) (Figure 7A). The heterogeneity among studies was low, with a tau^2^ of 0 (95%-CI: 0.00–0.98), a tau of 0 (95%-CI: 0.00–0.99), an I^2^ of 0% (95%-CI: 0–79%), and an H of 1.00 (95%-CI: 1.00–2.19) (Figure 7A). The Q statistic was 3.47 with four degrees of freedom and *p*-value of 0.48, indicating that the heterogeneity was not statistically significant (Figure 7A). The funnel plot in Figure 7B is relatively symmetrical, with most studies concentrated in the middle and lower part of the graph. This suggests that there is no significant publication bias or heterogeneity among the studies. However, one study by Sangouni et al. (7) is located at the bottom of the plot, indicating a large and negative effect size with low precision.

### 3.8 Summary of findings of meta analysis

To formally assess the certainty of our findings, we applied the GRADE (Grading of Recommendations Assessment, Development and Evaluation) framework, and the results are presented in Table 3. Our analysis indicates a high certainty of evidence for the significant reduction in BMI, supported by a low heterogeneity (I^2^ = 0%) and a precise effect estimate. In contrast, the evidence for a significant effect on lipid profiles varies. We found moderate certainty of evidence for the reduction in TC levels due to moderate heterogeneity (I^2^ = 37%), while the evidence for TG and LDL-C reductions was graded as low certainty due to high inconsistency and imprecision, respectively. The certainty of evidence for the effect on HDL-C was categorized as very low, primarily because of high heterogeneity (I^2^ = 83%) and a wide confidence interval that included no effect. These findings highlight the variable confidence in the pooled results for each metabolic parameter and underscore the influence of heterogeneity on the overall evidence quality.

**Table 3:** Summary of findings.

Outcome	Effect estimate (95% CI)	Certainty of evidence	Justification
TG levels	MD: −16.76 mg/dL (−29.44 to −4.09)	Low	Inconsistency: High heterogeneity (I^2^ = 77%). Imprecision: Wide confidence interval crossing the null effect in FEM.
TC levels	MD: −10.54 mg/dL (−4.64 to −16.45)	Moderate	Inconsistency: Moderate heterogeneity (I^2^ = 37%), but not statistically significant.
HDL-C levels	MD: −1.90 mg/dL (−4.24 to 0.43)	Very low	Inconsistency: High heterogeneity (I^2^ = 83%). Imprecision: Confidence interval includes no effect.
LDL-C levels	MD: −5.01 mg/dL (−1.22 to 11.23)	Low	Inconsistency: High heterogeneity (I^2^ = 67%). Imprecision: Confidence interval includes no effect.
BMI	MD: −0.94 kg/m^2^ (−0.68 to −1.20)	High	No serious concerns: Heterogeneity is low (I^2^ = 0%), and the confidence interval is narrow.

### 3.9 A clinical overview

Building on the growing interest in using nutraceuticals for managing metabolic health and childhood obesity (11), a number of recent clinical trials have investigated the specific effects of curcumin on lipid profiles. Studies have consistently demonstrated that curcumin, often in combination with bioavailability enhancers like piperine, can exert significant lipid-modifying effects in patients with conditions like metabolic syndrome and type 2 diabetes (12–14). Furthermore, research has explored the efficacy of curcumin as part of a multi-ingredient nutraceutical blend, noting its benefits on oral fat load in post-prandial states (15). However, the clinical effects of curcumin can vary with different formulations, as highlighted by a study showing that a highly bioavailable micellar form did not reduce blood lipids or inflammation markers in hyperlipidemic individuals (16). These varied findings from individual trials collectively underscore the importance of our meta-analysis, which provides a comprehensive, evidence-based synthesis of the overall efficacy of curcumin in managing key metabolic parameters like blood lipids and BMI (Table 3).

### 3.10 Safety

Based on the comprehensive analysis of clinical trials investigating curcumin supplementation in metabolic disorders, the safety profile appears to be generally favorable with minimal adverse effects reported across multiple studies. In the randomized controlled trial by Panahi et al. (10), curcuminoid supplementation at a dose of 1,000 mg/day for 12 weeks in type 2 diabetic patients demonstrated excellent tolerability with no serious adverse events reported, and the incidence of mild gastrointestinal discomfort was comparable to the placebo group. Similarly, the study by Adibian et al. (17) using 1,500 mg/day curcumin for 10 weeks in diabetic patients reported no significant adverse effects, with the treatment being well-tolerated and no dropouts due to side effects. The research by Adab et al. (6) utilizing 2,100 mg/day turmeric powder for 8 weeks in hyperlipidemic diabetic patients found no clinically significant adverse events, and laboratory safety parameters including liver and kidney function tests remained within normal ranges. In the trial by Thota et al. (5) combining curcumin with omega-3 fatty acids in prediabetic individuals, the supplementation was well-tolerated with no reported adverse effects on metabolic parameters or organ function. The nano-curcumin study by Rahimi et al. (18) also demonstrated good safety profile with no serious adverse events reported during the intervention period. However, some studies have noted mild and transient gastrointestinal symptoms including nausea, diarrhea, and abdominal discomfort in a small subset of participants, but these effects were generally self-limiting and did not require treatment discontinuation (19, 20). The long-term safety data from Karandish et al. (9) and Sangouni et al. (7) further support the favorable safety profile of curcumin supplementation, with no significant alterations in hematological or biochemical safety markers observed. Overall, the collective evidence from these clinical trials suggests that curcumin supplementation at doses ranging from 1,000–2,100 mg/day for periods of 8–12 weeks is generally safe and well-tolerated in patients with metabolic disorders, with the most common side effects being mild gastrointestinal symptoms that rarely lead to treatment withdrawal.

### 3.11 Risk of bias

**Figure 8:**
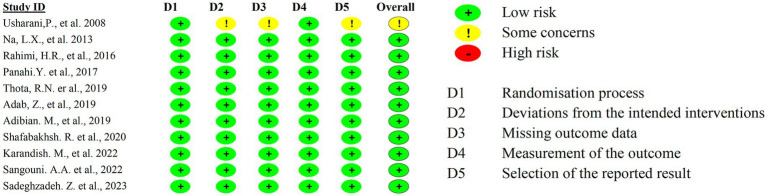
Risk of bias.

The included studies consistently demonstrate the beneficial effects of curcumin and its derivatives on metabolic parameters, including blood glucose regulation, lipid profile improvement, and inflammation reduction. Most studies reported reductions in triglycerides (TG), total cholesterol (TC), and low-density lipoprotein cholesterol (LDL-C), as well as improvements in insulin resistance. Additionally, some studies indicated reductions in body mass index (BMI) and inflammatory cytokines. Despite these promising findings, the potential for bias must be considered. All studies reported positive outcomes with equal weighting, raising concerns about publication bias or selective reporting (Figure 8). Furthermore, variations in study design, sample sizes, and control measures may introduce methodological inconsistencies. While some studies employed placebo controls, the extent of blinding and randomization methods was not consistently detailed. Given these limitations, the overall risk of bias remains a consideration in interpreting the findings. Although the consistency of reported benefits supports the therapeutic potential of curcumin, further large-scale, high-quality RCTs with rigorous methodologies are necessary to validate these effects and reduce the impact of potential biases.

## 4 Discussion

The meta-analysis investigated curcumin’s impact on blood lipid profiles and BMI in patients with metabolic diseases, revealing significant effects with notable heterogeneity. Curcumin supplementation notably reduced TG level, with a MD of 11.00 mg/dL in the FEM and 16.76 mg/dL in the REM. However, substantial heterogeneity (I^2^ = 77%) suggests variability across studies, which could arise from differences in study design, populations, and curcumin formulations. Baujat and Galbraith analyses identified studies, involving Adab et al. (6) and Sangouni et al. (7), as contributors to this heterogeneity due to their unique study populations and intervention characteristics.

For TC level, curcumin led to a significant reduction, with moderate heterogeneity (I^2^ = 37%), suggesting a more consistent effect than that for TG level. In contrast, curcumin’s effects on HDL-C and LDL-C levels were less consistent. A significant increase in HDL-C level was found in the FEM rather than in the REM, likely due to high heterogeneity (I^2^ = 83%), which could be potentially influenced by variability in demographics and dosages. Similarly, curcumin significantly reduced LDL-C level in the FEM rather than the REM, with moderate heterogeneity (I^2^ = 67%). These findings emphasize the importance of examining dosage, duration, and patient characteristics to better understand curcumin’s variable effects.

Moreover, the overrepresentation of studies from a single region (9 out of 11 trials were conducted in Iran) may introduce regional bias and limit the generalizability of our findings. Factors such as genetic background, dietary habits, and healthcare practices specific to the Iranian population could influence the response to curcumin supplementation. Future studies should include more diverse populations to validate these results across different ethnic and regional groups. However, it is important to acknowledge the limitations of publication bias assessment in meta-analyses with a small number of studies. Begg’s and Egger’s tests are known to have low statistical power when the number of included studies is limited, as in our analysis (*n* = 11). The presence of outliers, coupled with the absence of studies reporting negative or null results in the funnel plot, suggests that publication bias cannot be entirely ruled out. Therefore, our conclusion of no significant publication bias should be interpreted with caution. Future studies with larger sample sizes are needed to validate these findings.

The REM is more reliable for interpreting these findings as it accounts for between-study variability, providing more conservative effect size estimates and confidence intervals. In contrast, the FEM assumes a uniform effect size, which is less appropriate given the observed heterogeneity. Consequently, the FEM may overestimate the effect size and underestimate its confidence interval, potentially leading to false positive conclusions (21). In cases of high heterogeneity (e.g., HDL-C and LDL-C analyses), the random-effects model was prioritized to avoid overestimation of effects, as it accounts for between-study variance. The fixed-effects model was only applied when heterogeneity was low (e.g., TC and BMI analyses).

A deeper exploration of curcumin’s dosage and supplementation duration is warranted, as these factors may influence the heterogeneity observed. Studies with smaller sample sizes and shorter intervention durations, such as Adab et al. (6) and Sangouni et al. (7), highlighted the potential impact of these variables. For instance, Adab et al. (6) concentrated on a highly selective population (patients with type 2 diabetes and hyperlipidemia), whereas Sangouni et al. (7) included a broader group of patients with metabolic diseases, while had a brief intervention period. These differences underscore the need for subgroup analyses to elucidate the influence of study characteristics on curcumin’s effects.

Furthermore, the clinical significance of curcumin’s lipid-lowering effects should be emphasized alongside statistical significance. While the reductions in lipid levels are promising, their translation into long-term cardiovascular benefits remains unclear. Future research should concentrate on the practical implications of these findings, exploring whether the observed improvements in lipid profiles translate into reduced cardiovascular events or improved patient outcomes.

Curcumin, a natural polyphenol derived from turmeric, has demonstrated beneficial effects on lipid metabolism and cardiovascular health in animal models (22, 23). This is likely due to curcumin’s anti-inflammatory and antioxidant properties, which are hypothesized to play a crucial role in mitigating dyslipidemia associated with metabolic diseases (24). These mechanisms may reduce the chronic inflammation and oxidative stress central to dyslipidemia. However, variability in outcomes highlights the need for optimization of curcumin administration, including its formulation, dosage, and duration. Additionally, potential synergies between curcumin and conventional hypolipidemic drugs warrant further investigation to enhance treatment strategies for diabetic dyslipidemia.

This interest stems from its multifaceted effects on key molecular targets, particularly the AMPK and PPARγ signaling pathways, which are central to regulating lipid metabolism and energy homeostasis (25). Research indicates that curcumin effectively activates AMPK, a crucial cellular energy sensor, through mechanisms involving upstream kinases and reactive oxygen species (26). This activation promotes beneficial metabolic changes, including enhanced fatty acid oxidation by increasing CPT-1 expression, and suppressed lipogenesis by inhibiting ACC and SREBP-1c. Furthermore, curcumin’s cross-talk with the adiponectin/AMPK/SIRT1 pathway improves insulin sensitivity and energy expenditure (27). Concurrently, curcumin modulates the activity of PPARγ, a nuclear receptor vital for adipocyte differentiation and lipid storage. This modulation is context-dependent: in adipocytes, curcumin-loaded nanospheres suppress adipogenesis by modulating PPARγ phosphorylation (28), while in hepatic cells, it downregulates PPARγ to reduce lipid accumulation (29). Despite these promising mechanisms, native curcumin faces major clinical limitations due to its poor bioavailability, solubility, and rapid metabolism (30). To overcome these challenges, recent advances have focused on novel formulation strategies. For example, curcumin-loaded nanospheres have shown significantly greater anti-adipogenic activity (28), and combining curcumin with bioenhancers like piperine markedly improves its absorption and prolongs its presence in the body (19). This dual-pathway regulation, coupled with innovative delivery systems, positions curcumin as a valuable therapeutic candidate for a range of metabolic conditions, including NAFLD, obesity, and dyslipidemia (20).

Although the reduction in BMI (MD: −0.94 kg/m^2^) was statistically significant, its clinical relevance warrants careful interpretation. A decrease of less than 1 kg/m^2^ may not individually translate into substantial improvements in cardiovascular risk or diabetes control. However, even modest reductions in BMI at a population level can contribute to meaningful public health benefits, particularly when combined with improvements in other metabolic parameters such as triglycerides and total cholesterol. Future studies should directly assess the impact of curcumin on hard clinical endpoints, such as cardiovascular event rates or diabetes-related complications, to better define its therapeutic utility.

Despite the observed heterogeneity and variable outcomes in lipid profiles, the consistent beneficial effects of curcumin on TG, TC, and BMI underscore its potential as an adjunct therapy in metabolic disorders. The anti-inflammatory and antioxidant properties of curcumin may underlie these improvements, though further mechanistic studies are warranted to elucidate its precise modes of action. Future research should prioritize well-designed randomized controlled trials with standardized curcumin formulations, adequate dosing, longer durations, and diverse populations to enhance the generalizability and clinical applicability of findings. Moreover, investigating synergies between curcumin and conventional lipid-lowering therapies could provide valuable insights for combination treatment strategies. This therapeutic potential of curcumin is further supported by a recent meta-analysis of meta-analyses, which concluded that curcumin strongly and consistently decreases pain and improves joint mobility in patients with osteoarthritis (31).

Several limitations should be considered. First, the predominance of studies from Iran may limit generalizability to other populations. Second, variability in curcumin formulations, dosages, and intervention durations across studies could contribute to heterogeneity. Third, the potential for publication bias, though not statistically significant in funnel plot tests, cannot be entirely ruled out. Future research should include larger, multi-regional RCTs with standardized protocols to validate these findings.

## 5 Conclusion

Despite its potential, the predominance of Iranian studies limits the generalizability of these findings. The regional bias may affect the extrapolation of results to other populations due to potential differences in genetic, dietary, and lifestyle factors. Broader, globally representative research is essential to confirm curcumin’s efficacy across diverse populations and to standardize its use in clinical practice.

## Data Availability

The original contributions presented in the study are included in the article/supplementary material, further inquiries can be directed to the corresponding author.
